# Genomic stability among O3:K6 *V. parahaemolyticus* pandemic strains isolated between 1996 to 2012 in American countries

**DOI:** 10.1186/s12863-021-00985-0

**Published:** 2021-09-27

**Authors:** Abraham Guerrero, Bruno Gomez-Gil, Marcial Leonardo Lizarraga-Partida

**Affiliations:** 1 Cátedras CONACyT-CIAD, Food Research and Development Center A.C. Mazatlán Unit (Centro de Investigación en Alimentación y Desarrollo, A.C. Unidad Mazatlán), Mazatlán, Sinaloa Mexico; 2 CIAD, Food Research and Development Center A.C. Mazatlán Unit for Aquaculture, A.P. 711, Mazatlán, Sinaloa Mexico 82100; 3 Centro de Investigación Científica y de Educación Superior de Ensenada, Baja California (CICESE), Ensenada, Baja California Mexico

**Keywords:** *V. parahaemolyticus* outbreaks, *V. parahaemolyticus* in American countries, *V. parahaemolyticus* O3:K6 pandemic clone

## Abstract

**Background:**

The *V. parahaemolyticus* pandemic clone, results in the development of gastrointestinal illness in humans. Toxigenic strains of this species are frequently isolated from aquatic habitats and organisms such as mollusks and crustaceans. Reports on the isolation of the pandemic clone started in 1996, when a new O3:K6 clone was identified in Asia, that rapidly spread worldwide, becoming the predominant clone isolated from clinical cases. In this study whole genome sequencing was accomplished with an Illumina MiniSeq platform, upon six novel *V. parahaemolyticus* strains, that have been isolated in Mexico since 1998 and three representative genomes of strains that were isolated from reported outbreaks in other American countries, and were deposited in the GenBank. These nine genomes were compared against the reference sequence of the O3:K6 pandemic strain (RIMD 2210633), which was isolated in 1996, to determine sequence differences within American isolates and between years of isolation.

**Results:**

The results indicated that strains that were isolated at different times and from different countries, were highly genetically similar, among them as well as to the reference strain RIMD 2210633, indicating a high level of genetic stability among the strains from American countries between 1996 to 2012, without significant genetic changes relative to the reference strain RIMD 2210633, which was isolated in 1996 and was considered to be representative of a novel O3:K6 pandemic strain.

**Conclusions:**

The genomes of *V. parahaemolyticus* strains isolated from clinical and environmental sources in Mexico and other American countries, presented common characteristics that have been reported for RIMD 2210633 O3:K6 pandemic strain. The major variations that were registered in this study corresponded to genes non associated to virulence factors, which could be the result of adaptations to different environmental conditions. Nevertheless, results do not show a clear pattern with the year or locality where the strains were isolated, which is an indication of a genomic stability of the studied strains.

## Background

*V. parahaemolyticus* is a Gram-negative bacterium that is commonly distributed in marine environments. This bacterium has been associated with foodborne infections, causing 3 major syndromes: gastroenteritis, wound infections, and septicemia [1]. Most *V. parahaemolyticus* strains capable of causing infection express genes that encode thermostable direct hemolysin (*tdh*), *tdh*-related hemolysin (*trh*), or both. These genes have been identified as the primary virulence factors [2, 3] and are commonly used to identify pathogenic strains [4, 5].

Infections that are caused by *V. parahaemolyticus* in the US and Mexico have historically been associated with the O4:K12 serotype [6]; however, after the first outbreak of a novel O3:K6 clone (O3:K6/*tdh*^+^/*trh*^−^), reported in India in 1996, the O3:K6 clone spread throughout Southeast Asia and various geographical zones, including countries in the American continent, resulting in a pandemic event [7] and becoming the most common serotype that was associated with infection, globally [1].

The detection of this pandemic strain in American countries was first reported in Peru, in 1996; however, the first outbreak was registered in 1997–1998 [8, 9], and since then, outbreaks have been reported in other countries in the American continent [10]. The US recorded 416 infection cases in 1998, most of which were linked to the consumption of raw seafood (oysters) from the Gulf of Mexico [11]. In Chile, greater than 16,000 infection cases have been reported since 1998, representing the highest recorded number of infections [10, 12, 13]. Brazil presented the lowest incidence of infections, with only 18 clinical cases reported from 2001 to 2002 [14]. Guerrero et al. [15], found that the O3:K6 clinical strain had been isolated in Mexico by INDRE (Mexican Epidemiological Institute) between 1998 to 2009, in different states, and Revilla-Castellanos et al. [16] identified the strain in 2012 among hull biofouling samples from a ship with Japanese provenance. O3:K6 strains were also isolated in 2004, from the only recorded outbreak in Mexico, which resulted in more than 1200 clinical cases [17].

This study represents the first attempt to explore genomic variations among the pandemic *V. parahaemolyticus* O3:K6 strains isolated from 1998 to 2012 in Mexico and their genetic similarities to strains that have been isolated from other outbreaks in American and Asian countries (Table 1).

**Table 1:** List of studied O3:K6 strains isolated in American countries

Strain	Year	State	GenBank	Characteristics
CICESE-170*	1998	Hgo	JAABPG000000000	R72H, *tlh, tdh, orf8,* O3:K6 (+), *trh* (−)
CICESE-186*	1999	Hgo	JAABPH000000000	R72H, *tlh, tdh, orf8,* O3:K6 (+), *trh* (−)
CICESE-187*	2000	Tams	JAAIFK000000000	R72H, *tlh, tdh, orf8,* O3:K6 (+), *trh* (−)
CAIM 1400*	2004	Sin	JAAIFJ000000000	R72H, *tlh, tdh, orf8,* O3:K6 (+), *trh* (−)
CICESE-188*	2009	NL	JAAHBO000000000	R72H, *tlh, tdh, orf8,* O3:K6 (+), *trh* (−)
CICESE-273*	2012	BC	JAABPI000000000	R72H, *tlh, tdh, orf8,* O3:K6 (+), *trh* (−)
RIMD 2210633	1996	Japan	BA000031/BA000032	O3:K6, *tdh* (+), *trh* (−)
Peru-466	1996	Peru	ACFM00000000	O3:K6, *tdh* (+), *trh* (−)
CDC_K5058	2007	USA	MITP00000000	O3:K6, *tdh* (+), *trh* (−)
ATC210	1998	Chile	LFUN00000000	O3:K6, *orf 8*, *tdh* (+), *trh* (−)

The novel (*) strains isolated in Mexico at the states of Hidalgo (Hgo), Tamaulipas (Tams), Sinaloa (Sin), Nuevo Leon (NL) and Baja California (BC).

## Methods

### Collection of strains

*V. parahaemolyticus* strains, collected during various years and varying locations in Mexico, were selected for this study. The reference genomes of strains from outbreaks that were registered in Latin America and the US and the reference genome of the strain RIMD 2210633, isolated in Japan were also included as representative genomes of the O3:K6 pandemic clone. The list of strains is presented in Table 1.

### Sequencing

Six O3:K6 *V. parahaemolyticus* strains (Table 1), described as belonging to sequence type 3 (ST3) by multilocus sequence typing (MLST) [15], were sequenced for the present study. Genomic DNA from a single colony of each strain was extracted using the cetrimonium bromide (CTAB) method. The obtained DNA was sequenced using the Illumina MiniSeq platform (2 × 150-bp paired-end reads) at the Mazatlán Unit for Aquaculture and Environmental Management A.C. (CIAD-Mazátlan).

### Genome assembly

The reads for each strain were used to obtain the coverage depth, using SAMtools V1.2 [18] and BWA-MEM V0.7.12 [19], implemented with coverage.sh script developed by our team (https://github.com/cabraham03/coverage/blob/main/coverage.sh), and the metrics were visualized in Qualimap V2.2 [20]. Each genome was assembled using SPAdes V3.8 [21], the de novo assembly was performed with careful mode and -k 21,33,55,77,99,127 parameters. The resulting contigs were submitted to the Rapid Annotation Subsystem Technology (RAST [22];).

The pan- and core-genome plots were constructed based on the shared gene families of the 6 genomes described here and the 3 genomes of the O3:K6 pandemic strains isolated from outbreaks reported in South America, ATC210 (Chile, GenBank: LFUN00000000), Peru-466 (Peru, GenBank: ACFM00000000), North America CDC_K5058 (USA, GenBank: MITP00000000), and the genome from the reference strain RIMD 2210633 (GenBank: BA000031 and BA000032), which was isolated in Asia. The analyses were implemented as follows. Contigs of each genome were used to generate gene predictions, using Prokka V1.14.6 [23], and then Roary V3.12.0 [24], was implemented to obtain the pan-genome. Gene presence/absence matrices were visualized using roary_plots.py V1.01 (https://github.com/sanger-pathogens/Roary/tree/master/contrib/roary_plots). The predicted amino acid were generated with Prodigal V2.6.3 [25], and CMG-Biotools [26] was implemented to obtain a pairwise comparison with blastmatrix program. If a BLAST hit showed a 50% identity match in the alignment, and the length of the alignme nt was at least 50% of the longest gene (50/50 cut-off), it was considered to be a protein family.

### Comparative analysis

To visualize the similarities and differences between strains, contigs from each genome were assembled in 2 supercontigs for each chromosome (−I and -II), using the MeDuSa web server (http://combo.dbe.unifi.it/medusa; [27]), and aligned with blast ring image generator (BRIG, V0.95 [28];). The comparative analyses were focused on the primary mobile genetic elements that were associated with the pandemic strain, such as the 7 VPaIs, phage f237, super integron (SI), and secretion systems (TSS).

## Results

The number of reads for the obtained sequences ranged from 619,896 for CICESE-188 to 6,256,738 for CAIM 1400. The resulting reads presented average lengths of between 71 bp for CICESE-170 to 145 bp for CAIM 1400. The coverage depth showed differences between strains, wherein the lowest registered coverage was 14.2×, for CICESE-188, and the highest was registered for CAIM 1400, at 155.9×. Genomes were assembled from between 143 contigs (N50 = 194,688 and L50 = 8) for CICESE-170 to 1590 contigs (N50 = 5908 and L50 = 244) for CICESE-188 (Table 2). Among the Mexican genomes, CAIM 1400 presented the highest number of detected genes, with 4901 genes, including 283 new genes and 280 new gene families; CICESE-170 had the lowest number of genes, with 4643 detected genes, including 87 new genes and 69 new gene families (Table 2).

**Table 2:** Metrics obtained for the Mexican O3:K6 strains

Genome	No reads	bp	Coverage	GC %	Contigs	N50	L50	Total genes	New genes/families	Year
CAIM 1400	6,256,738	145	155.9X	45.2	180	204,016	7	4901	283/280	2004
CICESE-170	3,940,460	71	52.9X	45.3	143	194,688	8	4643	87/69	1998
CICESE-186	1,045,832	134	26.5X	45.4	464	25,261	58	4699	102/96	1999
CICESE-187	1,202,227	121	27.5X	45.5	760	16,608	94	4732	121/121	2000
CICESE-188	619,896	120	14.2X	46.0	1590	5908	244	4778	313/310	2009
CICESE-273	1,890,618	117	33.7X	45.6	934	10,218	147	4759	109/108	2012

bp = Average base pair. Year = year of isolation. N50 = minimum conting needed to cover 50% of the genome. L50 = number of contigs whose length sum makes up 50% of the genome size.

**Fig. 1:**
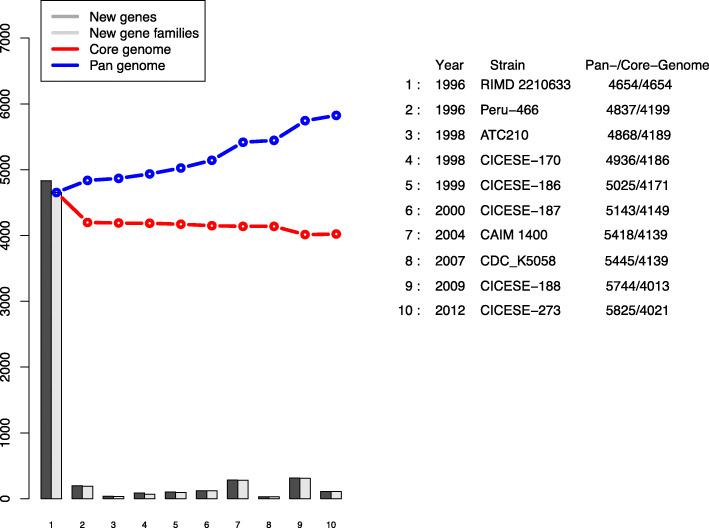
Pan- and core-genome plot of O3:K6 strains isolated in different American countries

The pan- and core-genomes, new genes and new gene families for each strain, are show in Fig. 1. The strains Peru-466, isolated in 1996, CAIM 1400, isolated in 2004, and CICESE-188, isolated in 2009, presented the most new genes and new gene families relative to the reference genome of the strain RIMD 2210633, which was isolated in 1996, without a clear pattern associated with the year of isolation. The pan-genome presented an increase in the number of genes, from 4654 genes in the RIMD 2210633 strain to 5825 genes that were registered for CICESE-273, an environmental strain isolated in 2012; in contrast, the core-genome decreased, from 4654 genes in RIMD 2210633 to 4013 in CICESE-188, which was isolated in 2009.

**Fig. 2:**
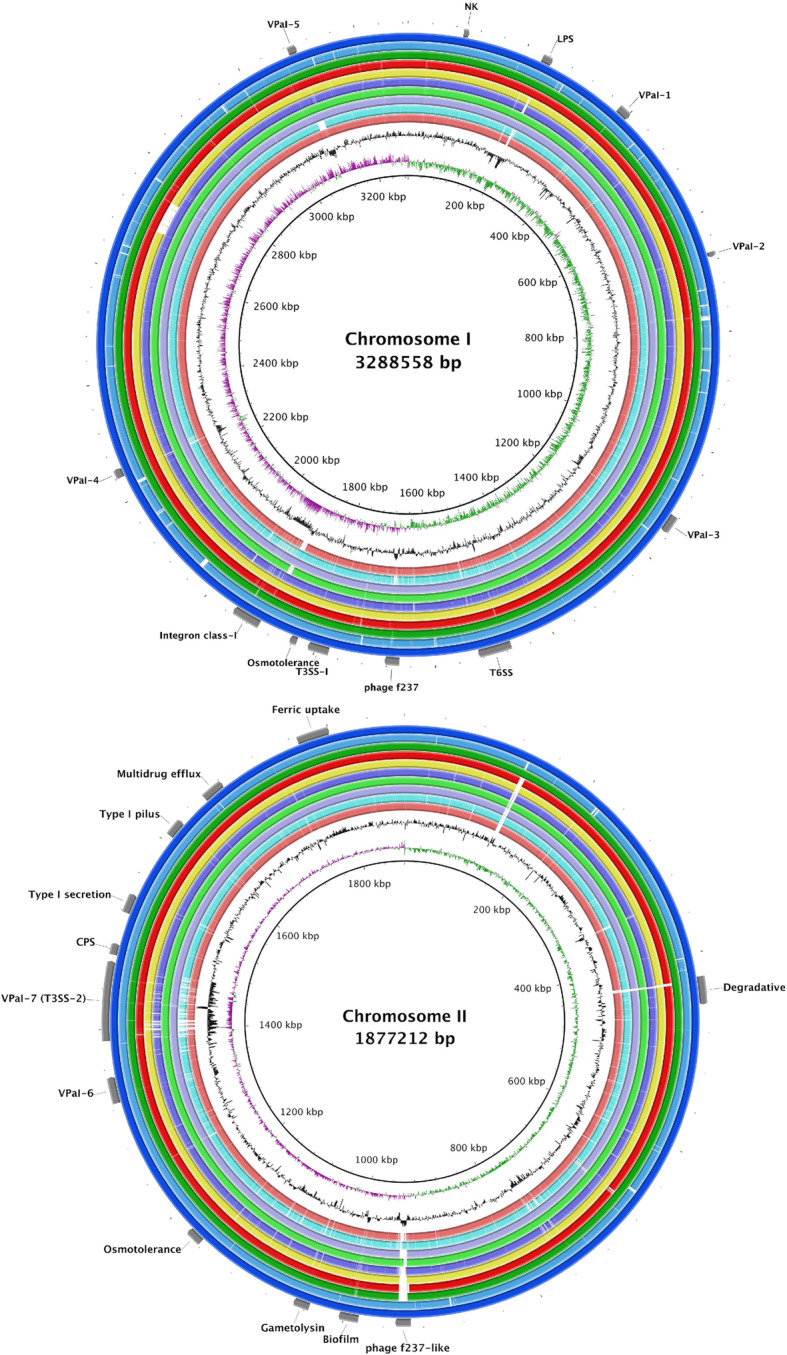
Alignment of the reference and novel genomes. From the outer ring to inner, RIMD 2210633 (, Japan), Peru-466 (, Peru), ATC210 (, Chile), CICESE-170 (  , Mexico), CICESE-186 (, Mexico), CICESE-187 (  , Mexico), CAIM 1400 (  , Mexico), CDC_K5058 (  , USA), CICESE-188 (  , Mexico), CICESE-273 (, Mexico), GC Content (). Most common elements associated to O3:K6 strains are indicated in the figure

The alignment of chromosomes I and II for the O3:K6 strains isolated in American countries, relative to the reference strain RIMD 2210633, which was isolated in Japan, are presented in Fig. 2. For both chromosomes, the figure indicates the most common genetic mobile elements that were associated with the pandemic strains, which were identified as the 7 VPaIs, the phage f237, the secretion system (TSS), and the type I pilus, among others. The positions of each element corresponded with those in the genome of the reference strain RIMD 2210633, as described in Hurley et al. [29], Boyd et al. [30], and Chen et al. [31].

From the RAST web server analysis, the genes commonly associated with pathogenic strains were detected, in categories such as resistance to antibiotics and toxic compounds, phages, prophages, iron acquisition systems, stress responses, toxins, the regulation of virulence genes, secretion systems, flagellar motility, capsular and extracellular polysaccharides, siderophores, colonization, and biofilm formation. Based on the RAST results, using a function-based comparison tool, most of the differences relative to the reference genome RIMD 2210633 were identified in genes that were associated with categories other than pathogenicity.

Mobile genetic elements that were associated with O3:K6 pandemic strains were detected in both chromosomes of the studied O3:K6 strains (Fig. 2). Five of the VPaIs (VPaI-1 to − 5) were detected in chromosome I, whereas the other 2 (VPaI-6 and -7) were detected in chromosome II. The results indicated that the VPaIs that were detected in the Mexican strains contained most of the genes that have been described for the reference genome RIMD 2210633, with high similarity (> 96.4%). The few variations that were identified were associated with non-coding bases.

The VPaIs 1, 2, 3, 4, and 6 were present in each genome of the Mexican strains, containing the same number of genes that were previously reported for RIMD 2210633. VPaI-5 was not detected in CICESE-188; however, this strain presented most of the mobile genetic elements, including transposase, hypothetical protein, lipopolysaccharides (LPS), phage f237, 2 secretion systems (T3SS-1 and − 2), an osmotolerance gene cluster, integron class I, type I pilus, a multidrug efflux gene cluster, ferric uptake, gametolysin, biofilm, degradative genes, and *tdh* gene, which are characteristic of pandemic strains. In VPaI-7, most of the Mexican genomes presented differences with the reference genome, in the genes *VPA1312*, *VPA1313*, *VPA1314*, *VPA1316*, *VPA1318*, and *VPA1357*. In addition, in most of the Mexican strains, the phage f237-like was not detected; a gap registered in chromosome II, associated with phage f237-like (6 o’clock), may be due to a close association with phage f237, which was registered in chromosome I.

**Fig. 3:**
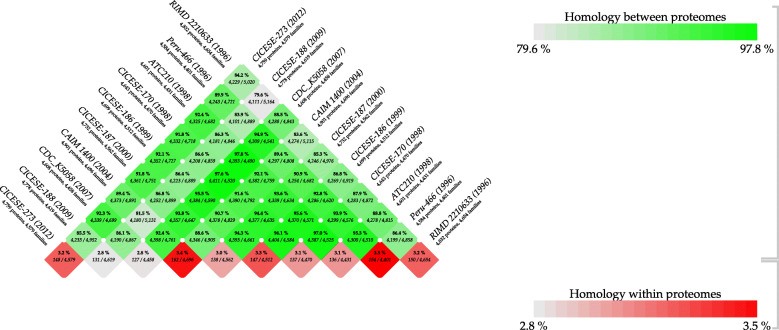
Predicted amino acids comparation between the Mexican genomes and the reference genomes obtained from outbreaks reported in America and Asia. Homology between predicted amino acids are represented in green and homology within predicted amino acids in red

Based on the BLAST matrix comparison, the homology between and within predicted amino acids is presented in Fig. 3. The analyzed genomes from strains isolated in various American countries registered a maximum of 4901 proteins within 4696 families, for CAIM 1400, whereas the reference strain RIMD 2210633 contained a total of 4832 proteins within 4654 families. The similarities in shared proteins between the American strains and RIMD 2210633 ranged from 79.6% (CICESE-188) to 88.8% (ATC210). The American strains presented higher percentages of similarities with each other, with CICESE-170 sharing 97.6% (4411) of proteins with CDC_K5058 and ATC210 sharing 97.8% (4393) of proteins with CDC_K5058. CICESE-188, isolated in 2009 in northeastern Mexico, presented the lowest percentage of shared proteins with all studied genomes. The homology among predicted amino acids ranged from 2.8 to 3.5%. Although no clear pattern was associated with isolation year, high percentages of shared proteins were registered between the Peru-466 strain, isolated in 1996, and the strains CICESE-170, isolated in Mexico in 1998, and ATC210, isolated in Chile in 1998, and these countries were the first American countries in which the pandemic clone was isolated.

**Fig. 4:**
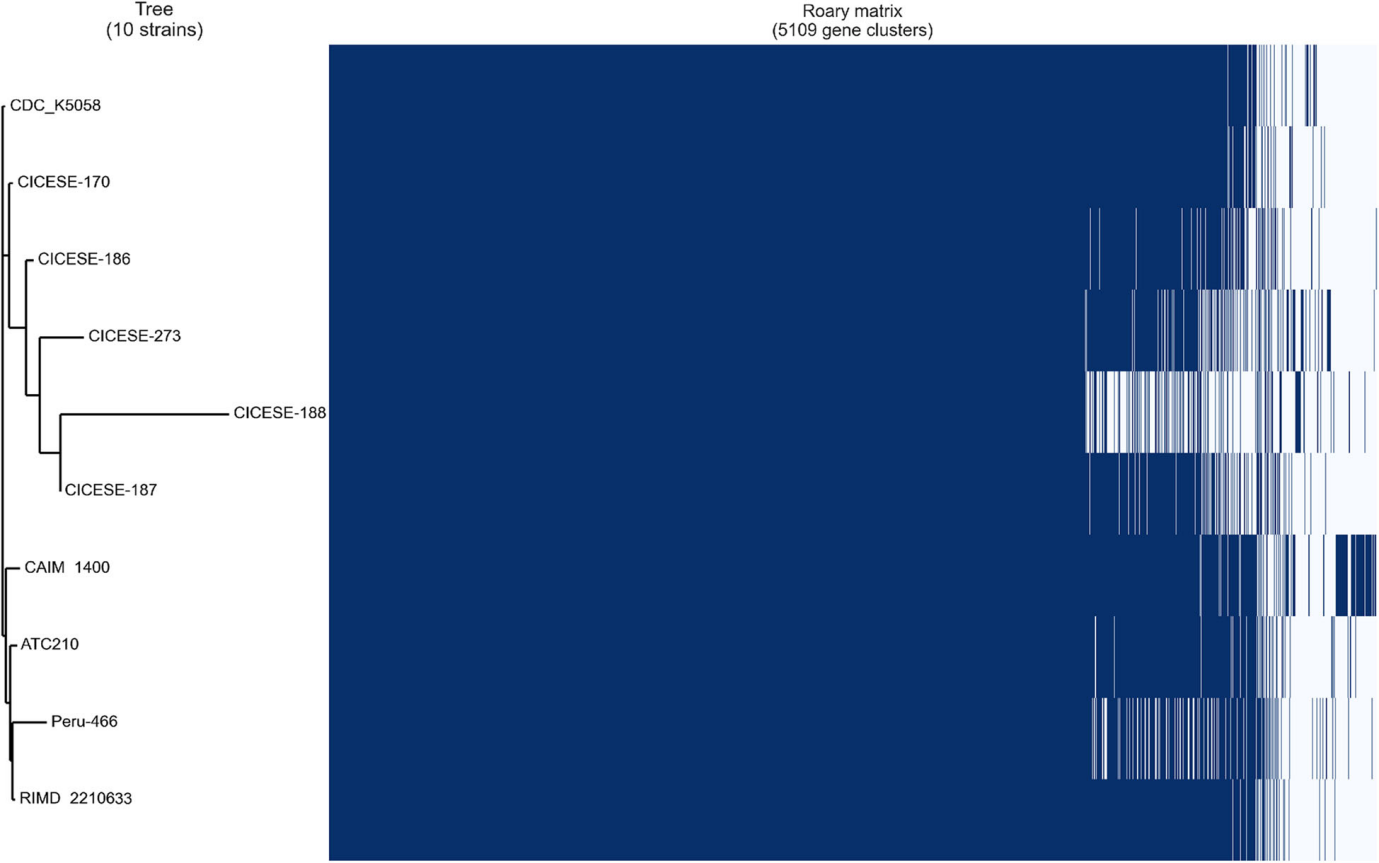
Dendogram based on a matrix of precense (dark blue) and absence (light blue) of genes and the distribution of the core and accessory genes of the 10 American *V. parahaemolyticus* O3:K6 studied genomes

The results presented in the pangenome of Fig. 4, indicated high similarity among the American O3:K6 strains and the reference strain RIMD 2210633. Two clusters could be distinguish in the dendrogram of this figure; one that groupe strains isolated in the USA in 2007 (CDC_K5058) and those isolated in Mexico in 1998 (CICESE-170), 1999 (CICESE-186), 2000 (CICESE-187) and 2009 (CICESE-188) and 2012 (CICESE-273). The strain CICESE-188, which was isolated in a northeastern state of Mexico, showed the lowest degree of similarity with the other strains in this group, and also displayed the lowest percentage of shared proteins. A second cluster was formed by those strains isolated in Peru (Peru-466) in 1996, in Chile (ATC 210) in 1998, CAIM 1400 isolated in 2004 in México and the reference strain RIMD 2210633 isolated in Japan in 1996.

## Discussion

The pandemic O3:K6 *V. parahaemolyticus* strains are closely related genetically and commonly clustered into a single group [32, 33]. These strains are genetically different from non-pandemic strains, including the old O3:K6 (*trh*+/*tdh*-) clone [7]. Molecular approaches have demonstrated that O3:K6 pandemic strains presented a clonal origin [34], in which most of the strains presented almost identical molecular patterns [35, 36] or the same sequences for *toxRS* [7]. ST3 was the most common MLST found among the pandemic strains [32, 37]. Based on these studies, the clonal term has been commonly used to describe O3:K6 pandemic strains. However, whole-genome analyses have detected differences between the closely genetically related strains, which could not be discovered by sequencing only a few genes (*toxRS* or MLST).

The present study included 6 Mexican strains, isolated from 1998 to 2012, most of which were isolated from clinical cases [15], and 3 strains that were isolated in American countries from 1996 to 2007. Differences were found between the numbers of new genes and new gene families, shared proteins, and phylogenetic relatedness (Figs. 1–4); however, the homology among predicted amino acids ranged from 79.6 to 97.8% (Fig. 3), which indicated that these strains are closely related genetically. Based on the genome analysis (Fig. 1), American O3:K6 strains presented new genes and new families of genes, which were not detected in the reference genome of the strain RIMD 2210633; most of these differences were associated with genes that have been classified as hypothetical proteins, with unknown function or with genes that are not necessary for infection.

These *V. parahaemolyticus* O3:K6 pandemic strains presented mobile genetic elements, such as the filamentous phage f237, which has been widely associated with this serotype [38]. Some of these mobile genetic elements (ORF8) have been used as molecular markers for pandemic strains [39]. However, the most common elements that were associated with the O3:K6 pandemic strains are the VPaIs [30, 33], which are mobile elements that harbor multiple putative virulent genes, encoding hydrolases, colicins, M proteins, cytotoxin integrase, methyltransferase (MTase), and the TDH toxin, as well as genes that are associated with the T3SS2 gene cluster and phage-like proteins [2, 29, 31, 40–42]. The presence of these elements has been correlated with pathogenicity [43]. Hurley et al. [29] hypothesized that these elements increase the fitness or the infectivity of O3:K6 pandemic strains. VPaI-1, VPaI-4, VPaI-5, and VPaI-6 have been reported to be found exclusively in O3:K6 pandemic strains [29, 30], in addition to the mobile elements in VPaI-7, in chromosome II. These elements encode several virulent factors, such as *tdh,* which encodes the TDH toxin, and the T3SS2, which has been implicated in host cell invasion [44]. These elements were detected in all of the American strains, with only a few genetic variations relative to the reference strain RIMD 2210633 (Fig. 2).

## Conclusions

These results showed that the genomes of *V. parahaemolyticus* strains isolated from clinical and environmental sources in Mexico and other American countries presented common characteristics that have been reported for the O3:K6 RIMD 2210633 pandemic strain, and the major variations that were registered in this study corresponded with genes that have been categorized as non-pathogenic, which could be the result of adaptations that were necessary for the different environmental conditions of the localities from which they were isolated [45]. However, the studied genomes did not present any clear patterns according to the year or region of isolation, based on pan/core genomes, shared proteins, and phylogenetic relatedness, which is concerning. Therefore, the results from these *V. parahaemolyticus* pandemic strains indicated a high level of genetic stability among the strains from American countries between 1996 to 2012, without significant genetic changes relative to the reference strain RIMD 2210633, which was isolated in 1996 and was considered to be representative of a novel O3:K6 pandemic strain. The pangenome presented in Fig. 4, show the association of strains isolated in the North of the continent as well as the association of those strains isolated in the South of the continent, with the exception of CAIM 1400 that was isolated in 2012, during the only outbreak registered in México. Nevertheless, results do not show a clear pattern with the year or locality where the strains were isolated, which is an indication of a genomic stability of the studied strains.

## Data Availability

The genome sequences of Mexican *V. parahaemolyticus* O3:K6 strains were deposited at GenBank, under accession numbers JAABPG000000000 (CICESE-170), JAABPH000000000 (CICESE-186), JAABPI000000000 (CICESE-273), JAAIFJ000000000 (CAIM 1400), JAAIFK000000000 (CICESE-187), and JAAHBO000000000 (CICESE-188), as genome submission SUB6862244.

## Abbreviations

CIAD: Centro de Investigación en Alimentación y Desarrollo, A.C. Unidad Mazatlán
CICESE: Centro de Investigación Científica y de Educación Superior de Ensenada, Baja California
CONACyT: Consejo Nacional de Ciencia y Tecnología
*tdh*: thermostable direct hemolysin
*trh*: *tdh*-related hemolysin
MLST: Multilocus sequence typing
RAST: Rapid Annotation Subsystem Technology
VPal: *V. parahaemolyticus* pathogenicity Island
CAIM: Collection of Aquatic Important Microrganisms (CIAD)
